# Mechanism of action of quercetin in regulating cellular autophagy in multiple organs of Goto-Kakizaki rats through the PI3K/Akt/mTOR pathway

**DOI:** 10.3389/fmed.2024.1442071

**Published:** 2024-08-15

**Authors:** Zhiqun Guo, Jingyu Zhang, Mianxin Li, Zengwei Xing, Xi Li, Jiaqi Qing, Yuan Zhang, Lemei Zhu, Mingxu Qi, Xuemin Zou

**Affiliations:** ^1^Hunan Key Laboratory of the Research and Development of Novel Pharmaceutical Preparations, Changsha, China; ^2^School of Public Health, Changsha Medical University, Changsha, China; ^3^Department of Cardiovascular Medicine, Affiliated Nanhua Hospital, University of South China, Hengyang, Hunan, China

**Keywords:** quercetin, autophagy, diabetes mellitus, GK rat, PI3K/AkT/mTOR pathway

## Abstract

**Objective:**

This experimental study investigated the protective function of quercetin on the liver, spleen, and kidneys of Goto-Kakizaki (GK) rats and explores its mechanism of action on autophagy-related factors and pathways.

**Materials and methods:**

GK rats were randomly divided into three groups: DM, DM + L-Que, and DM + H-Que, with age-matched Wistar rats serving as the control group. The control and DM groups were gavaged with saline, and the quercetin-treated group was gavaged with quercetin for 8 weeks each. Weekly blood glucose levels were monitored. Upon conclusion of the experiment, blood samples were gathered for lipid and hepatic and renal function analyses. The histopathologic morphology and lipid deposition in rats were examined. Disease-related targets were identified using molecular docking methods and network pharmacology analysis. Subsequently, immunohistochemical analysis was performed, followed by Western blotting to evaluate the levels of autophagy-related proteins and proteins in the AKT/PI3K/mTOR pathway, as well as their phosphorylation levels.

**Results:**

The results showed that, compared with the control group, the DM group exhibited significant increases in blood glucose, serum liver and kidney markers, liver fat vacuoles, and inflammatory cell infiltration. Immunohistochemistry (IHC) results indicated that quercetin reduced the extensive expression of AKT, P62, and mTOR in the liver and spleen of diabetic rats. The expression of autophagy and pathway-related proteins, such as P62, PI3K, P-PI3K, Akt, P-AKT, mTOR, and P-mTOR, was upregulated, while the expression of LC3A/LC3B, Beclin-1, Pink-1, and Parkin was downregulated. Conversely, the quercetin group showed a reduction in liver and kidney injury serum markers by decreasing lipid deposition and cell necrosis, indicating that quercetin has protective effects on the liver, spleen, and kidneys of GK rats. Additionally, in the quercetin group, the expression of autophagy and pathway-related proteins such as LC3A/LC3B, Beclin-1, Pink-1, and Parkin was upregulated, while the expression of P62, PI3K, P-PI3K, Akt, P-AKT, mTOR, and P-mTOR was downregulated, with statistically significant correlations.

**Conclusion:**

Quercetin markedly ameliorates liver, spleen, and kidney damage in GK rats, potentially through the inhibition of the PI3K/Akt/mTOR pathway, promoting autophagy. This research offers a rationale to the therapeutic potential of quercetin in mitigating organ damage associated with diabetes.

## 1 Introduction

A metabolic disorder characterized by abnormally high levels of glucose in the blood is called diabetes mellitus (DM) (1–3). To date, the treatment of diabetes remains a significant focus of human research. The most recent information available from the International Diabetes Federation, 463 million adults worldwide have diabetes (4). Nevertheless, the disease’s control rate continues to be suboptimal, highlighting a substantial challenge that necessitates the implementation of efficacious strategies for its prevention and management. Prolonged dysregulation of glucose and lipid metabolism leads to detrimental effects on several organs, notably the liver and kidneys (5, 6), spleen (7) and other solid organs.

The liver and kidneys play crucial roles in the body’s glucose metabolism. Individuals with diabetes frequently suffer from liver impairment as a consequence of metabolic dysregulation (8, 9). Kidney damage, accompanied by a marked escalation in the prevalence of diabetes mellitus (DM) among clinical patients, has resulted in a notable increase in the incidence of DM-associated liver and kidney impairments. The spleen plays a crucial role as an immune organ within the human body (10, 11). Individuals diagnosed with diabetes exhibit diminished immune capabilities, adversely impacting the spleen and leading to its impairment. In Traditional Chinese Medicine (12) suggests that liver damage associated with Diabetes Mellitus (DM) is intricately linked to the liver and kidneys’ physiological roles in nourishment and Qi transformation. From a physiological perspective, there is a mutual support mechanism between the liver and spleen (13). The primary manifestations are twofold. Initially, the liver and spleen mutually enhance their functions, aiding both the absorption and digestion of nutrients and the metabolism of fluids. Subsequently, these organs collaborate to facilitate the circulation of Qi and blood throughout the body. This physiological synergy implies that any pathological changes in one organ can reciprocally affect the other. Consequently, considering the conditions of deficiency or excess, it is postulated that damage to the liver, spleen, and kidneys is interlinked.

Quercetin (Que), a flavonoid found (14) in diverse fruits and vegetables such as apples, grapes, citrus fruits, cherries, various teas, red wine, cauliflower, and green vegetables (15). Quercetin has a variety of biological functions. The biological functions of quercetin include antidiabetic, anti-inflammatory, antioxidant, and anticancer effects (16). Despite its efficacy in diabetes management, the precise mechanisms underlying its action are not well understood. Consequently, this investigation adopts a comprehensive research methodology, integrating network pharmacology (17) and molecular docking, to perform an in-depth analysis of the ‘Traditional Chinese Medicine-Component-Target’ relationship.

Cellular autophagy not only regulates lipid metabolism (18), but also inhibits oxidative stress, inflammation, and ameliorates apoptosis (19, 20). In mammals, autophagy is integral to glucose and lipid metabolism regulation, essential for growth, development, cell differentiation, and response to stress. It significantly downregulates mTOR expression, thus inhibiting cell proliferation, with mTOR being key in cell cycle and proliferation regulation (21). Additionally, Phosphatidylinositol 3-kinase (PI3K) serves as a critical signal in autophagy regulation within eukaryotes (22). The PI3K/Akt/mTOR pathway is instrumental in managing the release of inflammatory factors, oxidative stress, cell apoptosis, and autophagy, notably affecting autophagy in diabetes mellitus (DM) cells. AKT, a pivotal effector within the signaling cascade, is inducedly activated through the mediation of upstream PI3K, which in turn activates the downstream mTOR pathway. The mTOR pathway encompasses two distinct complexes, mTORC 1 and mTORC 2. mTORC 2, upon activation, directly phosphorylates its upstream target, AKT, effectuating the full activation of AKT’s kinase activity. Autophagy offers both preventive and therapeutic benefits for liver, kidney, and spleen damage induced by diabetes. However, the role of quercetin, particularly through mitochondrial autophagy in protecting these organs, remains unexplored. Thus, the present study utilized a comprehensive analysis of network pharmacology (23) and molecular docking (24) to assess the dynamic relationship of “herbal medicine-ingredient-target” with the aim of determining whether quercetin reverses autophagy inhibition in diabetic GK rats via the PI3K/Akt/mTOR pathway.

This investigation leverages the GK rat model to mimic type II diabetes, examining quercetin’s protective effects on the liver, kidneys, and spleen in diabetic conditions and its influence on the interplay between these organs. Its goal is to furnish a theoretical framework for addressing organ damage induced by diabetes. Employing network pharmacology and molecular docking, the research thoroughly explores the “Traditional Chinese Medicine-Component-Target” relationships, with the objective of determining if quercetin promotes autophagy in the liver, kidneys, and spleen of GK rats by targeting the PI3K/Akt/mTOR pathway, thereby unveiling innovative approaches and targets for diabetes management. This study provides valuable new perspectives and research directions for the treatment of diabetes.

## 2 Materials and methods

### 2.1 Experimental animals

Twelve-week-old male Goto-Kakizaki (GK) and Wistar rats were acquired from Changzhou Cavens Experimental Animal Co., Ltd. and Hunan SLAC Jingda Experimental Animal Co., Ltd., respectively. Prior to their housing in a specific pathogen-free (SPF) facility, the rats underwent a one-week acclimatization period. The experimental conditions included exposure to natural daylight, maintaining a constant temperature of 22°C, and a relative humidity between 40 and 70%. Rats were provided with both a high-fat and standard pellet diet and had unrestricted access to water and food. The Hunan Provincial Institute of Traditional Chinese Medicine’s Animal Ethics Committee granted approval for all experimental procedures (Approval No. 2019-0041, dated March 19, 2020).

### 2.2 Experimental equipment

Glucose test strips (purchased from Sinocare Inc.), Jinwen Blood Glucose Meter (purchased from Sinocare Inc.), Electronic Analytical Balance (purchased from Mettler-Toledo), Pipettors (purchased from Dlong Scientific Instruments Co., Ltd.), −80°C Ultra-Low Temperature Freezer (purchased from Zhongke Meiling Cryogenic Technology Co., Ltd.), Compact High-Speed Refrigerated Centrifuge (purchased from Eppendorf), Chemiluminescent Imaging Analysis System (purchased from Bio-Rad, Singapore), Protein Vertical Electrophoresis and Transfer System, Electrophoresis Tank, Electrophoretic Transfer Cell (purchased from Bio-Rad, Singapore), Horizontal Shaker (Beijing Liuyi Biotechnology Co., Ltd.), Tissue Grinder Homogenizer (Tiangen Biotech Co., Ltd.). Tissue Grinder Homogenizer (Tiangen Biotech Co., Ltd.), Dehydrator (purchased from Changzhou Zhongwei Electronic Instrument Co., Ltd.), Embedding Machine (purchased from Changzhou Zhongwei Electronic Instrument Co., Ltd.), Pathological Microtome (purchased from Thermo Fisher Scientific Inc.), Freezing Table (purchased from Wuhan Junjie Electronics Co., Ltd.), Water Bath-Slide Drier (purchased from Changzhou Zhongwei Electronic Instrument Co., Ltd.), Adhesion Microscope Slides (purchased from HuBei BIOSSCI Biotechnology Co., Ltd.), Upright Microscope (purchased from Olympus Corporation Inc.), Imaging System (purchased from Hamamatsu Photonics Corporation of Japan), Pipette Gun (purchased from DLAB Scientific Co., Ltd.), Pap Pen (purchased from HuZhou Maixin Biotechnology Co., Ltd.).

### 2.3 Experimental reagents

Quercetin (purity >90%) was purchased from Nanjing Spring & Autumn Biological Co., Ltd.; paraformaldehyde (4%) was obtained from Sigma Biological Technology Co., Ltd.; 10 mmol/L PBS buffer and TBST were acquired from New Cell American Bio-Technology Co., Ltd.; gel casting kits were bought from Changsha Dingguo Biotechnology Co., Ltd., Western blotting related antibodies were procured from Cell Signaling Technology and Hangzhou Hua’an Biotechnology Co., Ltd., and the ECL chemiluminescence kit was purchased from GLPBIO, United States. Anhydrous ethanol was purchased from Sinopharm Chemical Reagent Co., Ltd.; 30%H2O2 was purchased from Sinopharm Chemical Reagent Co., Ltd.; Normal Goat Serum was purchased from WuHan Boster Biological Technology Co., Ltd.; DAB Substrate Kit was purchased from HuZhou Maixin Biotechnology Co., Ltd.; Immunohistochemical related antibodies was purchased from UpingBio Technology Co., Ltd.; Hang Zhou, China and Proteintech Group Co., Ltd.; WuHang, China.

### 2.4 Experimental grouping and administration of drugs

**Table 1:** Detailed table of subgroups.

Groups	Quantities	Medicines	Dosages (mg/kg)	Mode of administration	Duration of administration
DM	10	Saline	5	Gastric lavage	8 weeks
Control	10	Saline	5	Gastric lavage	8 weeks
DM + L-Que	10	Quercetin	50	Gastric lavage	8 weeks
DM + H-Que	10	Quercetin	75	Gastric lavage	8 weeks

Twelve-week-old GK rats were given a high-fat diet for ten weeks in a row. GK rats with fasting blood glucose levels >7.1 mmol/L were considered successful models of diabetes. Subsequently, the GK rats were randomly divided into 3 groups (see Table 1).

Quercetin was administered orally to the subjects using a 5% carboxymethylcellulose sodium solution. The control group comprised Wistar rats receiving a 0.9% saline solution. Over the course of 8 weeks, each group received consistent oral administrations. Observations and recordings of body weight, overall health status, and food consumption were systematically conducted. Upon concluding the study, liver tissues were harvested from the GK rats.

### 2.5 Specimen collection and preparation

Following an 8-week regimen of pharmacological treatment, GK rats underwent an 8-h fast, after which their blood glucose levels and fasting body weight were assessed. The day’s requisite anesthesia dosage was determined by body weight, utilizing 10% pentobarbital sodium at a rate of 0.4 mL/100 g, and was subsequently administered. After the spleen is removed, weighed, liver, spleen, and kidney samples are removed, cleaned, and fixed in formaldehyde solution for future paraffin sectioning; additionally, a segment was preserved in cryovials, rapidly frozen in liquid nitrogen, and stored at −80°C for impending Western blot analysis. To safeguard integrity, specimens were maintained on ice throughout the collection phase. Blood samples were centrifuged within 12 h at 3,500 rpm for 10 min, and the isolated serum was kept in 1.5 mL EP tubes at −80°C for subsequent serum marker assessments.

### 2.6 Detection indicators

#### 2.6.1 Measurement of blood glucose levels

Weekly, following a 6-h fast, blood samples are collected from the tail tips of rats in each group and analyzed for glucose levels using test strips and a glucometer.

#### 2.6.2 Measurement of serum GSP, AST, ALT, TC, TG, TP, CHOL, UREA levels

1. Sample processing and requirements: serum: blood was taken from abdominal aorta in a test tube, after the blood was naturally coagulated, it was put into a centrifuge at 3,500 rpm and centrifuged for about 10 min, and the supernatant was taken for spare.
2. Analytical method: serum GSP, AST, ALT, TC, TG, TP, CHOL, and UREA were detected by automatic biochemical analyzer.

#### 2.6.3 Morphological observations

Previously formaldehyde-fixed liver, spleen, and kidney tissue sections were paraffin-embedded and stained with hematoxylin and eosin (H&E). Furthermore, liver sections underwent dehydration, cryosectioning, and Oil Red O staining to assess lipid droplet accumulation and pathological conditions microscopically.

#### 2.6.4 Immunohistochemistry stain

Liver and spleen tissues were fixed in 4% paraformaldehyde for over 24 h. Subsequently, the fixed tissues underwent antigen retrieval. The sections were incubated overnight at 4°C with the primary antibody working solution, followed by incubation with the secondary antibody working solution for 45 min after washing with TBST. Finally, the sections were stained with DAB chromogen and hematoxylin, dehydrated, and cover-mounted.

#### 2.6.5 Protein immunoblotting (Western blotting) detection

The detection of Beclin-1, LC3A, P62, Parkin, and Pink-1 protein expression in liver and spleen tissues from various groups was achieved through protein immunoblotting (Western blotting). Initially, liver and spleen tissues were sequentially processed to extract the total protein using a precise volume of protein lysis solution. This step was followed by electrophoresis and subsequent transfer of proteins onto a polyvinylidene fluoride (PVDF) membrane. The process concluded with the proteins being incubated with primary and secondary antibodies before fluorescence analysis was performed utilizing a photosensitive substrate.

### 2.7 The potential therapeutic applications of quercetin in diabetes prevention are elucidated through predictive analysis

**Figure 1:**
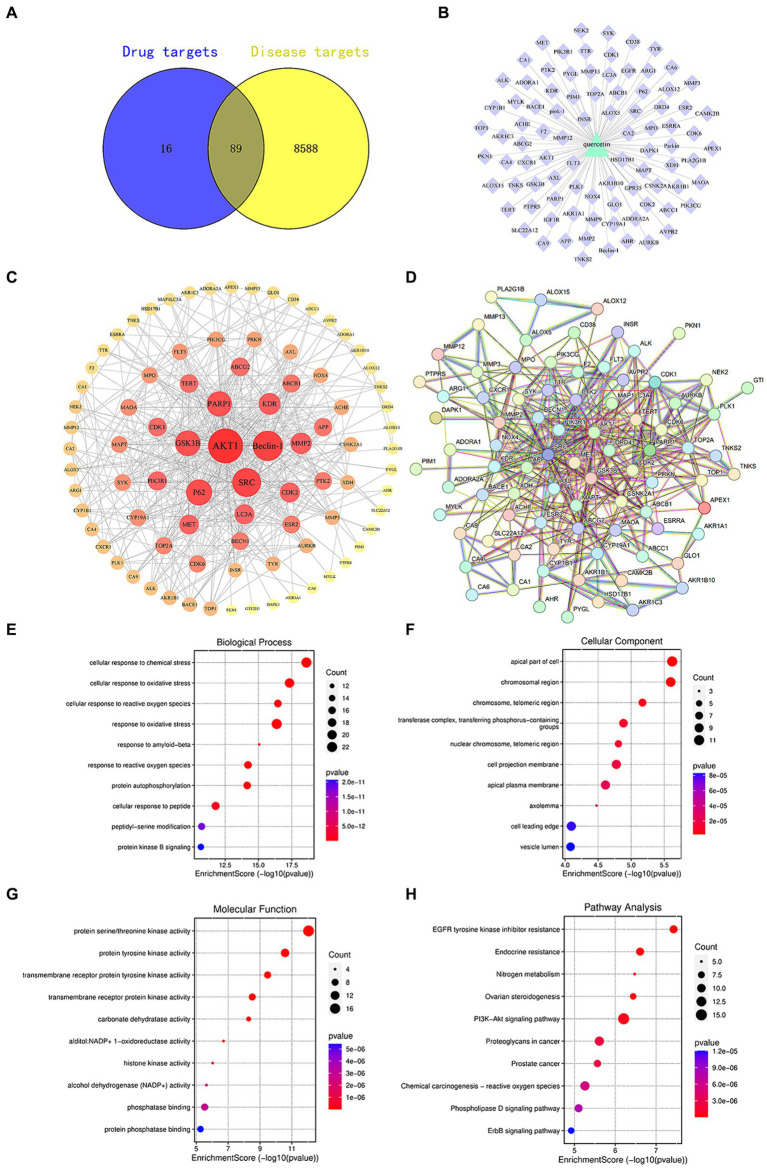
**(A)** Venn diagram of quercetin targets and diabetes targets. **(B)** Component-target diagram of quercetin targets and diabetes targets. **(C)** PPI network diagram. **(D)** Core target diagram. **(E)** Degree ranking graph of the top 10 core proteins in the PPI. **(F)** Bubble chart of the key target BP, CC, MF enrichment analysis for quercetin in the prevention of diabetes. **(G)** Bar chart of KEGG signaling pathway enrichment analysis. **(H)** Network diagram of quercetin treatment targets for diabetes—KEGG enrichment pathways.

Active compounds, such as quercetin, exert their pharmacological effects by modulating the expression of specific target genes or proteins, thereby intervening in pathological processes and offering therapeutic benefits for certain diseases. Utilizing the SwissTargetPrediction database, we identified 105 targets for quercetin. Subsequent searches in databases like OMIM and GeneCards revealed 8,677 targets associated with diabetes. A comparative analysis between quercetin’s targets and those linked to diabetes highlighted 89 core targets, as illustrated in Figure 1.

### 2.8 Quercetin target screening for diabetes treatment

Using “diabetes mellitus” as a keyword, we searched databases such as GeneCards,^1^ OMIM,^2^ DrugBank,^3^ TTD,^4^ and PharmGkb^5^ to identify targets related to DM (diabetes mellitus). Based on this, we used R software version 4.3.1 to intersect these targets with those identified in section 1.1 and created a Venn diagram to screen for the targets of quercetin’s intervention in DM.

### 2.9 Construction and analysis of the quercetin component-target network

The active components of quercetin and targets for DM intervention were imported into Cytoscape (v3.8.0 software) to draw the quercetin component-target network diagram. Topological structure analysis was carried out using the Network Analysis function to obtain degree value information.

### 2.10 Statistical analysis

The data obtained from the experiments were statistically analyzed and plotted using GraphPad Prism 9.5 software, represented as mean ± standard deviation (SD). Comparisons between two groups were conducted using independent samples *t*-tests, whereas comparisons among several groups were conducted using one-way analysis of variance (one-way ANOVA). Multiple comparisons between weekly glycemic changes were made using a two-way ANOVA (two-way ANOVA). Differences were considered statistically significant at *p* < 0.05.

## 3 Results

### 3.1 Network pharmacology

#### 3.1.1 Identification of quercetin’s potential targets for diabetes prevention

Target sites of active components are defined as genes or proteins that are pivotal for the pharmacological effects of these components. By modulating the expression of these targets, active components can intervene in pathological processes or ameliorate diseases. Utilizing the SwissTargetPrediction database, 105 target sites were identified for quercetin. Further analysis, leveraging databases such as OMIM and GeneCards, revealed 8,677 potential targets associated with diabetes. The analysis of the overlap between quercetin’s targets and those associated with diabetes identified 89 core potential targets (Figure 1A). An analysis of the PPI network topology concerning the 89 potential core targets (Figure 1B) pinpointed AKT, LC3A, P62, 和 Beclin-1 (Figure 1C) as the quintessential targets for quercetin’s diabetes prevention strategy. Of these, the AKT1 was the first of 89 potential core targets (Table 2).

**Table 2:** Top 15 targets ranked by degree method in PPI network.

Number	Protein name	Degree
1	AKT1	53
2	Beclin-1	43
3	SRC	40
4	P62	34
5	GSK3B	33
6	PARP1	32
7	KDR	25
8	MMP2	24
9	CDK2	21
10	LC3A	21
11	MET	20
12	PIK3R1	19
13	CDK1	18
14	TERT	18
15	ABCG2	18

Using R4.0.3 software, we loaded the “enrichplot” and “org.Hs.eg.db” packages to run the enrichGO function, analyzing the GO enrichment of 89 key targets of quercetin in the treatment of diabetes mellitus. The obtained *p*-values were corrected using FDR with a *Q*-value threshold of ≤0.05, considering GO terms meeting this criterion as significantly enriched. A total of 3,019 entries for biological process (BP), 270 entries for cellular component (CC), and 374 entries for molecular function (MF) were obtained. The top 10 entries of each enriched process were visualized using bubble plots (Figures 1E–G). The GO functional enrichment analysis indicated that the mechanism of diabetes prevention by quercetin is closely related to biological processes, especially cellular responses to chemical and oxidative stress. Subsequently, the “enrichKEGG” function in R4.0.3 was used to analyze the KEGG pathway enrichment annotations for the diabetes treatment targets of quercetin. After correcting the *p*-values using FDR, 214 pathways were identified with a *Q*-value threshold of ≤0.05. The top 10 pathways were visualized using bubble plots (Figure 1H). The KEGG pathway enrichment analysis elucidated that quercetin mainly affects signaling pathways, including PI3K-AKT and phospholipase D signaling pathways, creating a comprehensive, multi-targeted, and multifaceted pathway synergy that effectively regulates the body’s immune response and thus prevents diabetes.

### 3.2 Molecular docking

**Figure 2:**
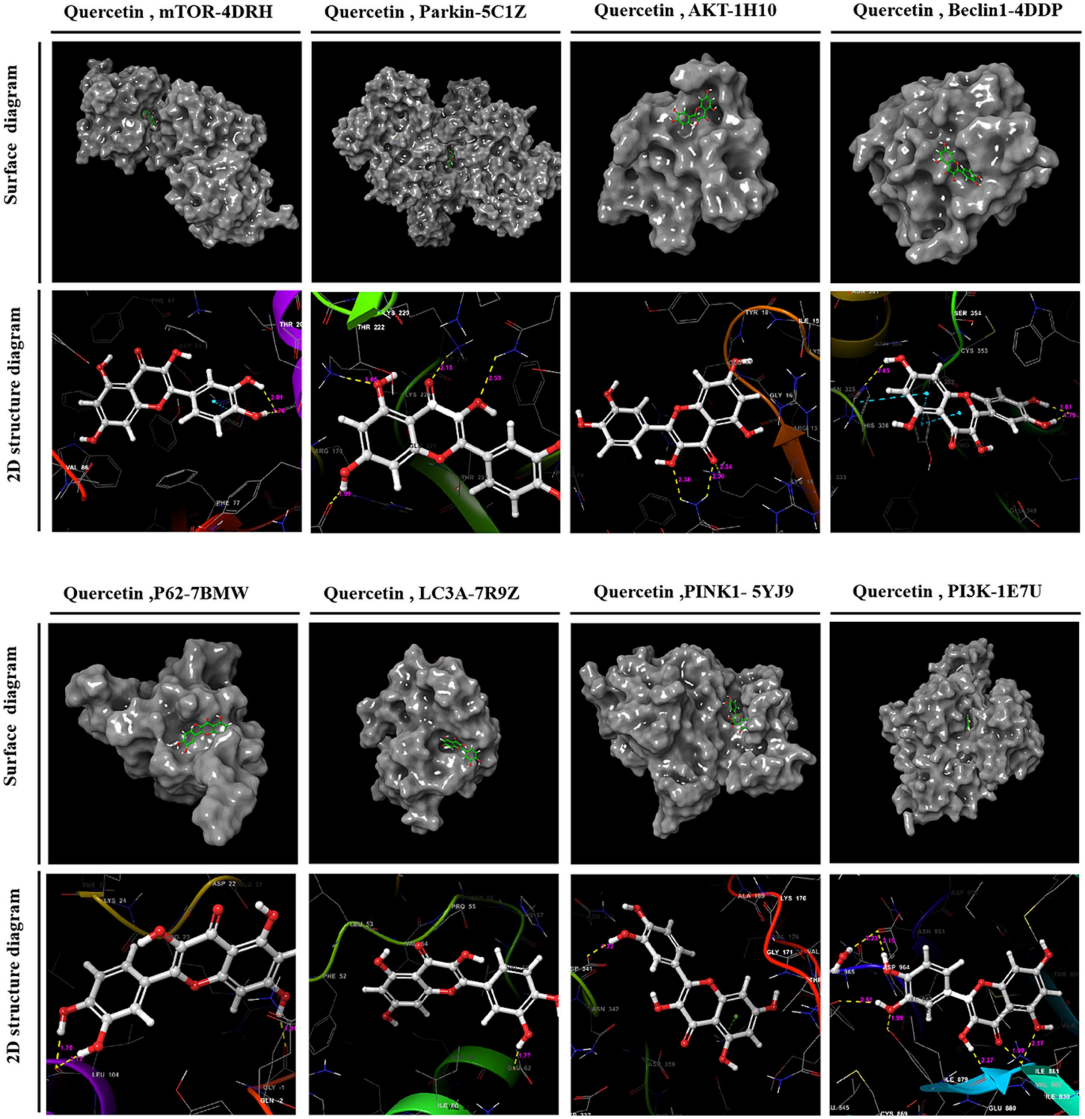
2D and 3D structural images of the complex formed by docking quercetin to its target.

Utilizing PPI topology analysis, mTOR, Parkin, AKT, Beclin-1, P62, LC3A, Pink-1, and PI3K were established as key targets for diabetes treatment. Typically, a lower binding energy signifies a more stable complex between a ligand and its receptor. Molecular docking revealed that quercetin forms stable complexes with these targets. Figure 2 illustrates the 2D and 3D structures of these quercetin-target complexes. The binding energies of these dockings are as follows: −6.766 kcal/mol for mTOR, −5.284 kcal/mol for Parkin, −4.478 kcal/mol for AKT, −5.447 kcal/mol for Beclin-1, −6.212 kcal/mol for P62, −4.609 kcal/mol for LC3A, −5.744 kcal/mol for Pink-1, and −8.333 kcal/mol for PI3K (Table 3), indicating significant stability and binding efficacy. The most stable interaction occurred with the PI3K protein, showcasing a docking binding energy of −8.333 kcal/mol. Analysis of the structure demonstrates quercetin’s tight binding within PI3K’s active site, facilitated by hydrogen bonds with amino acid residues IL3881, GLU880, TYR867, ASP841, and ILE963 at distances of 2.57 Å, 1.99 Å, 2.27 Å, 1.99 Å, 2.51 Å, and 2.15 Å, respectively. This evidence suggests quercetin’s capability to form tight, stable complexes with identified diabetes treatment targets, corroborating network pharmacology predictions regarding quercetin and its pivotal role in diabetes management.

**Table 3:** List of docking integration capabilities.

Target protein	Active ingredient	Binding energy (kcal/mol)
mTOR	Quercetin	−6.766
Parkin	Quercetin	−5.284
AKT	Quercetin	−4.478
Beclin-1	Quercetin	−5.447
P62	Quercetin	−6.212
LC3A	Quercetin	−4.609
Pink-1	Quercetin	−5.744
PI3K	Quercetin	−8.333

### 3.3 Blood glucose and body weight variations

GK rats exhibiting fasting blood glucose levels above 7.1 mmol/L were allocated randomly into three distinct groups: a diabetes model group (DM), a group receiving low-dose quercetin (DM + L-Que; 50 mg/kg/day), and a group receiving high-dose quercetin (DM + H-Que; 75 mg/kg/day). Prior to the initiation of quercetin treatment, no apparent differences were observed in the blood glucose values among the groups, although all exhibited levels considerably higher than those of the control group. Subsequent to oral administration, the DM + L-Que group demonstrated reduced blood glucose levels compared to the GK group, maintaining stability in glucose fluctuations. More pronounced, the DM + H-Que group exhibited a significant reduction in glucose levels, with a clear downward trend, in contrast to the GK group, which saw a continual rise in glucose levels.

**Figure 3:**
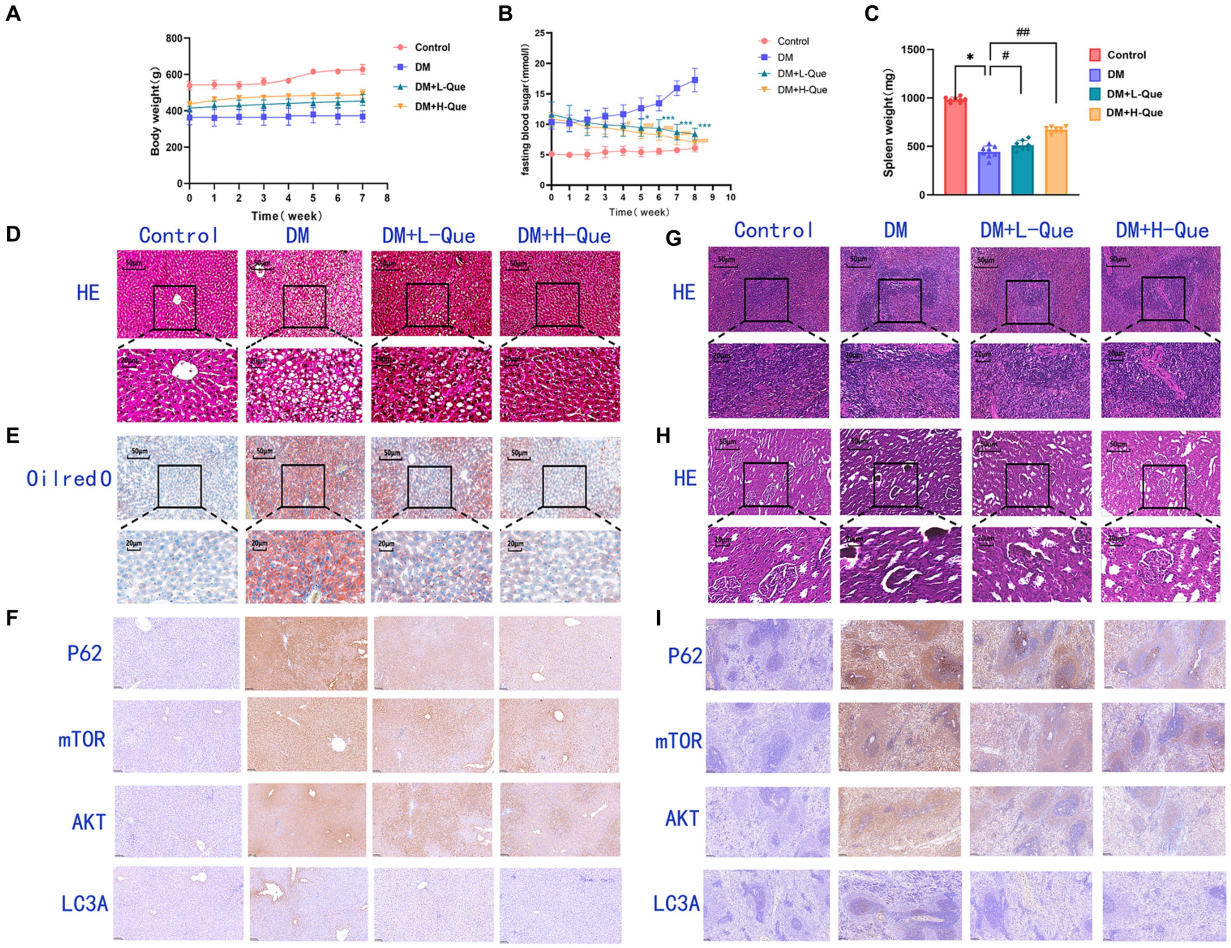
Effect of quercetin on the treatment of GK rats. **(A)** Body weight in each group after administration of quercetin or saline (*n* = 8). **(B)** Changes in blood glucose in each group after administration of quercetin or saline (*n* = 8). ^*^p < 0.05, ^**^p < 0.01, and ^***^p < 0.001 for DM + L-Que group and DM group; ^#^*p* < 0.05, ^##^*p* < 0.01, and ^###^*p* < 0.001 for DM + H-Que group and DM group. **(C)** Spleen weight in each group after administration of quercetin or saline (*n* = 8). ^*^p < 0.05, ^**^p < 0.01, and ^***^p < 0.001 for DM + L-Que group and DM group; ^#^*p* < 0.05, ^##^*p* < 0.01, and ^###^*p* < 0.001 for DM + H-Que group and DM group. **(D)** Significant effects of quercetin on structure and function in GK rats. Hematoxylin-eosin (HE) staining of liver sections in each group. Scale bar: 50 μm. **(E)** Oil red O staining of liver sections in each group. Scale bar: 50 μm. **(F)** *H*-score was used to calculate the liver positivity rate, and the expression of autophagy pathway AKT, LC3A, P62, and m-TOR was analyzed by IHC. Scale bar: 100 μm. IHC staining of AKT, LC3A, P62, and m-TOR expression was quantified using *H*-score = Σ*p_i_* (*i* + 1), where *p_i_* represents the number of positive cells as a percentage of the number of all cells in the section; *i* represents the intensity of coloring. **(G)** Hematoxylin-eosin (HE) staining of spleen sections in each group. Significant effects of quercetin on the structure and function of the spleen in GK rats. Scale bar: 50 μm. **(H)** Hematoxylin-eosin (HE) staining of kidney sections in each group. Significant effects of quercetin on renal structure and function in GK rats. Scale bar: 50 μm. **(I)** Spleen positivity was calculated using *H*-score, and autophagy pathway AKT, LC3A, P62, and m-TOR expression was analyzed by IHC. Scale bar: 100 μm. IHC staining of AKT, LC3A, P62, and m-TOR expression was quantified using *H*-score = Σ*p_i_* (*i* + 1), where *p_i_* represents the number of positive cells as a percentage of the number of all cells in the section; *i* represents the intensity of coloring.

Figure 3A illustrates the blood glucose level alterations across the groups. Moreover, quercetin positively influenced the body weights of diabetic rats, as depicted in Figure 3B. Initially, the body weight discrepancy between the quercetin-treated groups and the GK group was statistically insignificant. However, over time, the GK group’s weight increment was markedly milder compared to the control group. Quercetin also positively affected spleen weight in diabetic rats, as shown in Figure 3C. There was a statistically significant difference in spleen weight between the quercetin-treated groups and the GK group.

These findings affirm quercetin’s efficacy in lowering blood glucose levels and alleviating diabetes-induced weight loss in GK rats, suggesting its potential to improve the overall condition of diabetic patients.

### 3.4 Serum glycated protein levels

Table 4 presents the influence of quercetin on glycated serum proteins in rats. Glycosylated serum protein concentrations exhibited a significant increase in the DM group of GK rats compared to the control group (*p* < 0.01), highlighting inferior blood glucose regulation in these rats. Relative to the model group, treatment with quercetin in both the DM + L-Que group (diabetes with low-dose quercetin) and the DM + H-Que group (diabetes with high-dose quercetin) markedly enhanced the levels of glycated serum proteins in GK rats. Notably, the DM + H-Que group exhibited superior outcomes compared to the DM + L-Que group. These findings imply that quercetin contributes positively to the regulation of blood glucose in rats.

**Table 4:** Expression of GSP.

Group	GSP (mmol/L)
Control	218.67 ± 5.69
DM	293.33 ± 22.81^**^
DM + L-Que	261.00 ± 2.65^*^
DM + H-Que	244.33 ± 7.57^##^

Each value represents the mean = S.D. (*n* = 8). Data are regarded statistically significant with ^**^p < 0.01, compared to a control group; ^*^p < 0.05, compared to a control group; ^##^*p* < 0.01, comparison with model group; ^#^*p* < 0.05, comparison with model group, and ns >0.05 shows no difference group. GSP, good supply practice.

The effects of quercetin on lipid metabolism in GK rats, detailed in Table 5, indicate that serum levels of total cholesterol (TC) and triglycerides (TG) in the DM group were significantly higher than those in the control group (*p* < 0.01), highlighting lipid metabolic disorders in these rats. Compared to the DM group, treatment with quercetin in both the low-dose (DM + L-Que) and high-dose (DM + H-Que) protection groups resulted in a significant decrease in serum TC and TG levels (*p* < 0.01). This demonstrates quercetin’s capacity to ameliorate lipid metabolic irregularities and lower blood lipid levels in GK rats.

**Table 5:** Expression of TC and TG.

Group	TC (mmol/L)	TG (mmol/L)
Control	2.85 ± 0.13	0.39 ± 0.11
DM	8.96 ± 0.55^**^	1.98 ± 0.34^**^
DM + L-Que	5.57 ± 0.68^**##^	1.26 ± 0.18^**##^
DM + H-Que	4.69 ± 0.48^**##^	0.74 ± 0.17^**##^

Each value represents the mean = S.D. (*n* = 8). Data are regarded statistically significant with ^**^p < 0.01, compared to the control group; ^*^p < 0.05, compared to the control group; ^##^*p* < 0.01, comparison with model group; ^#^*p* < 0.05, comparison with model group, and ns >0.05 shows no difference group. TC, total cholesterol; TG, triglyceride.

Table 6 illustrates the impact of quercetin on liver function among GK rats. Serum concentrations of ALT (alanine aminotransferase) and AST (aspartate aminotransferase) in the diabetic (DM) group were Significantly higher than those in the control group (*p* < 0.01), suggesting compromised liver functionality. Compared with the model group, treatments with quercetin at low doses (DM + L-Que) and high doses (DM + H-Que) correspondingly mitigated ALT and AST levels, indicating an improvement in liver health. Notably, the high-dose quercetin group (DM + H-Que) demonstrated greater effectiveness than its low-dose counterpart (DM + L-Que).

**Table 6:** Expression of ALT and AST.

Group	ALT (U/L)	AST (U/L)
Control	35.71 ± 0.61	38.71 ± 0.61
DM	101.16 ± 23.96^**^	114.44 ± 16.89^**^
DM + L-Que	51.57 ± 0.59^##^	88.77 ± 2.72^**#^
DM + H-Que	43.14 ± 2.72^##^	75.48 ± 2.54^**##^

Each value represents the mean = S.D. (*n* = 8). Data are regarded statistically significant with ^**^p < 0.01, compared to the control group; ^*^p < 0.05, compared to the control group; ^##^*p* < 0.01, comparison with model group; ^#^*p* < 0.05, comparison with model group, and ns >0.05 shows no difference group. AST, aspartate aminotransferase; ALT, alanine aminotransferase.

The blood levels of total protein (TP), cholesterol (CHOL), and urea (UREA) were significantly higher in the DM group rats compared to the control group (*p* < 0.01), underscoring diabetes’ substantial impact on renal damage. This connection is further evidenced by the noted influence of kidney damage on the spleen. Subsequent to administering both high and low doses of quercetin, a decline in the concentrations of TP, CHOL, and UREA was recorded. Specifically, compared to the DM group, the high-dose quercetin treatment lead to a significant reduction in UREA levels by 47.20% (*p* < 0.01), as detailed in Table 7. Furthermore, this group also demonstrated decreases of 7.59% in TP and 37.02% in CHOL (*p* < 0.05 for both), as indicated in Table 7.

**Table 7:** Expression of TP, CHOL, and UREA.

Group	CHOL (mmol/L)	UREA (mg/dL)	TP (g/L)
Control	1.44 ± 0.21	6.70 ± 1.80	57.73 ± 1.57
DM	2.62 ± 0.30^**^	15.15 ± 1.51^**^	68.66 ± 2.15^**^
DM + L-Que	1.47 ± 0.04^##^	9.57 ± 1.14^##^	64.79 ± 0.61^**#^
DM + H-Que	1.65 ± 0.27^##^	8.00 ± 0.81^##^	63.45 ± 0.68^**#^

Each value represents the mean = S.D. (*n* = 8). Data are regarded statistically significant with ^**^p < 0.01, comparison with the control group; ^*^p < 0.05, comparison with the control group; ^##^*p* < 0.01, comparison with model group; ^#^*p* < 0.05, comparison with model group. CHOL, cholesterol; TG, triglyceride; UREA, urea nitrogen; TP, total serum protein.

### 3.5 Effect of quercetin intervention on histopathology of liver, spleen, and kidney

#### 3.5.1 Survey of hepatic hematoxylin and eosin staining

Figure 3D illustrates disrupted hepatic tissue architecture in diabetic GK rats, characterized by irregular hepatic lobule and hepatocyte arrangement and loosened cytoplasm. Cell nuclei varied in size and were often clustered, with a prevalence of fatty degeneration evident through numerous vacuolated cells in the observed field. Consequently, GK rats had dramatically elevated HE-staining lesions in the liver, marking severe steatohepatitis. Contrastingly, the quercetin-treated groups evidenced hepatocytes in a more organized arrangement, significantly ameliorating vacuolation and fatty degeneration. Notably, there was no significant inflammatory infiltration in the DM + H-Que group compared to the DM + L-Que group, along with conspicuously lower liver lesions.

Figure 3E further reveals, through Oil Red O staining, extensive red areas in diabetic GK rat sections, signifying pronounced lipid accumulation. Post-quercetin treatment, there was a significant diminution in liver lipid droplets.

#### 3.5.2 Investigations of hemoglobin and eosin staining of the spleen and kidneys

In the control group, rat spleen tissue exhibited a normal structure, with corpuscles shaped either round or oval, accompanied by arterial lymphatic sheaths adjacent to them. The ratio of white to red pulp was appropriate, featuring distinct demarcations. Conversely, DM group rats showed disorganized demarcation between spleen’s red and white pulp, diminished white pulp volume, attenuated arterial lymphatic sheaths, and an abundance of hemosiderophages. In rats treated with low-dose quercetin (DM + L-Que), the clarity of red and white pulp structures improved, with arterial lymphatic sheaths being slightly thicker than in the DM group, denser lymphocyte arrangements, and a thickening marginal zone. For the high-dose quercetin group (DM + H-Que), the spleen’s red and white pulp structures showed signs of normalization, with increasingly thicker arterial lymphatic sheaths and a lymphocyte arrangement that more closely resembled the control group. The data clearly indicates that spleen tissue structural damage in DM group rats progressively intensified, whereas the DM + L-Que and DM + H-Que treatments mitigated this damage, with the DM + H-Que group showing a significant trend towards control group levels (Figure 3G).

The control group’s renal tissue exhibited pristine structural integrity, with no evidence of vacuolar or granular degeneration in the tubular epithelial cells. Conversely, tubular epithelial cells within the DM group presented pronounced vacuolar and granular degeneration. Treatment with quercetin markedly enhanced renal tissue architecture, effectively mitigating both vacuolar and granular degeneration in tubular epithelial cells. Notably, the high-dose quercetin group (DM + H-Que) achieved superior amelioration compared to the low-dose group (DM + L-Que) (Figure 3H).

### 3.6 Comparative analysis of immunohistochemistry in different groups of GK rats

#### 3.6.1 Immunohistochemical expression in liver tissues of GK rats: overview of p62, LC3A, AKT, and mTOR proteins

In immunohistochemically stained sections, AKT is predominantly expressed in the hepatocyte membranes in the liver. The percentage area of hepatocyte membranes containing AKT protein is higher in the DM group (98.08%) compared to the control group. Compared to the DM group, the percentage area of hepatocyte membranes containing AKT protein is lower in the DM + L-Que group (90.78%) and significantly lower in the DM + H-Que group (88.44%) (Figure 3F). mTOR is extensively expressed in liver tissue, primarily in the cytoplasm. The percentage area of hepatocyte cytoplasm containing mTOR protein is higher in the DM group (98.08%) compared to the control group. Compared to the DM group, the percentage area of hepatocyte cytoplasm containing mTOR protein is lower in the DM + L-Que group (93.55%) and in the DM + H-Que group (92.33%) (Figure 3F). P62 is extensively expressed in liver tissue, primarily in the cytoplasm. The percentage area of hepatocyte cytoplasm containing P62 protein is higher in the DM group (98.94%) compared to the control group. Compared to the DM group, the percentage area of hepatocyte cytoplasm containing P62 protein is lower in the DM + L-Que group (91.93%) and significantly lower in the DM + H-Que group (78.69%) (Figure 3F). LC3 is expressed at low levels in the liver with a low positive rate, but the positive rate in the DM group (17.76%) is significantly higher than in the control, DM + L-Que, and DM + H-Que groups, primarily expressed in the cytoplasm of hepatocytes around the tissue edges and vascular areas (Figure 3F).

#### 3.6.2 Immunohistochemical expression in spleen tissues of GK rats: overview of p62, LC3A, AKT, and mTOR proteins

In immunohistochemically stained sections, splenic tissues showed predominantly membrane-expressed lymphocytes and scattered lymphocytes around the white pulp. The proportion of AKT-positive splenocytes mostly distributed in the cell membrane was elevated in the model group compared to the control group (73.22%). The proportion of AKT positive splenocytes was reduced in the low dose group (60.55%) as compared to the model group, whereas it was significantly reduced in the high dose group (28.47%) (Figure 3I). Similarly, the proportion of P62-positive splenocytes mostly distributed in the cytoplasm was elevated in the model group (98.03%) as compared to the control group. The percentage of P62-positive splenocytes mostly distributed in the cytoplasm was decreased in the low-dose group (91.21%), whereas the percentage was even lower in the high-dose group (64.6%) (Figure 3I). The percentage of mTOR-positive splenocytes mostly distributed in the cytoplasm was also higher in the model group compared to the control group (97.6%). In contrast, the percentage of mTOR-positive splenocytes was lower in the low-dose group (84.88%), whereas it was significantly lower in the high-dose group (80.02%) (Figure 3I). In addition, a higher proportion of LC3A-positive splenocytes were found in the model group (39.33%) compared to the control group. The proportion of LC3A-positive splenocytes was lower in the low-dose group (12.71%), whereas the proportion of LC3A-positive splenocytes was significantly lower in the high-dose group (3.60%) (Figure 3I).

### 3.7 Differential expression of autophagy-related pathway proteins in GK rats among various experimental groups

#### 3.7.1 Autophagy-related proteins expression in the hepatic tissues of GK rats: an overview of Beclin-1, p62, LC3A/LC3B, Pink-1, and Parkin proteins

**Figure 4:**
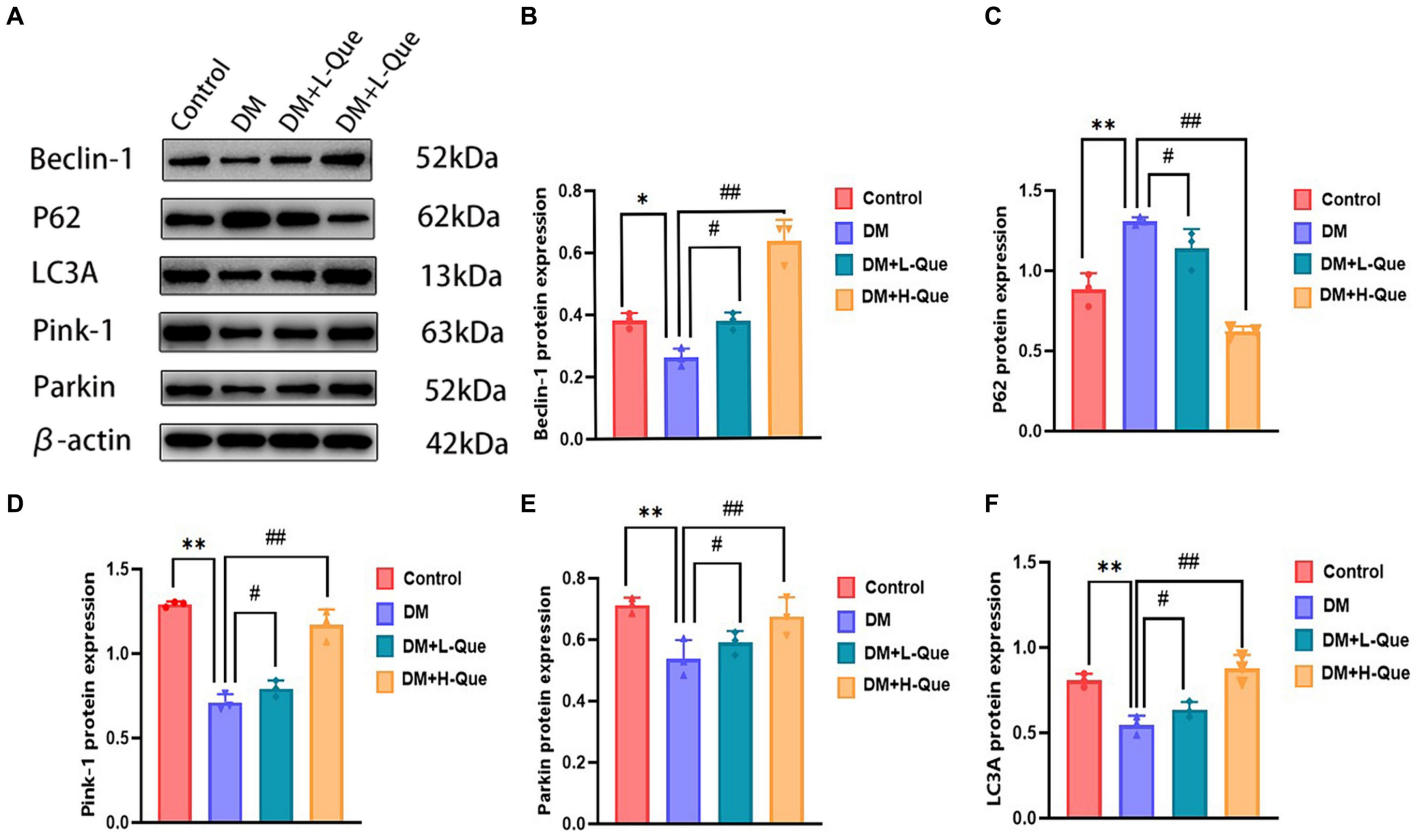
Quercetin promotes autophagy to ameliorate liver injury in GK rats. **(A)** Western blot gel images of liver tissue in each group (*n* = 8). **(B–F)** The bar graphs represent the quantification of gel bands (Expression of Beclin1, P62, LC3A, Pink1, and Parkin, mean ± SD). ^**^p < 0.01, comparison with the control group; ^*^p < 0.05, comparison with the control group; ^##^*p* < 0.01, comparison with model group; ^#^*p* < 0.05, comparison with model group.

According to the results of the Western blot analysis, the GK rats in the model group had a hepatic expression of P62 that was significantly higher (*p* < 0.01), whereas the expression levels of Beclin-1, LC3A/LC3B, Pink-1, and Parkin demonstrated a notable decrease (*p* < 0.01). In contrast, within the quercetin-protected groups, DM + L-Que and DM + H-Que, a significant reduction in P62 expression (*p* < 0.01) was observed in the liver tissues of GK rats, alongside a marked increase in the manifestation of Beclin-1, LC3A/LC3B, Pink-1, and Parkin proteins (*p* < 0.01). This indicates the protective role of quercetin on the hepatic function of diabetic GK rats through the promotion of autophagy (Figure 4).

#### 3.7.2 Autophagy-related protein expression in GK rat spleen tissues: a comparative study across various groups

**Figure 5:**
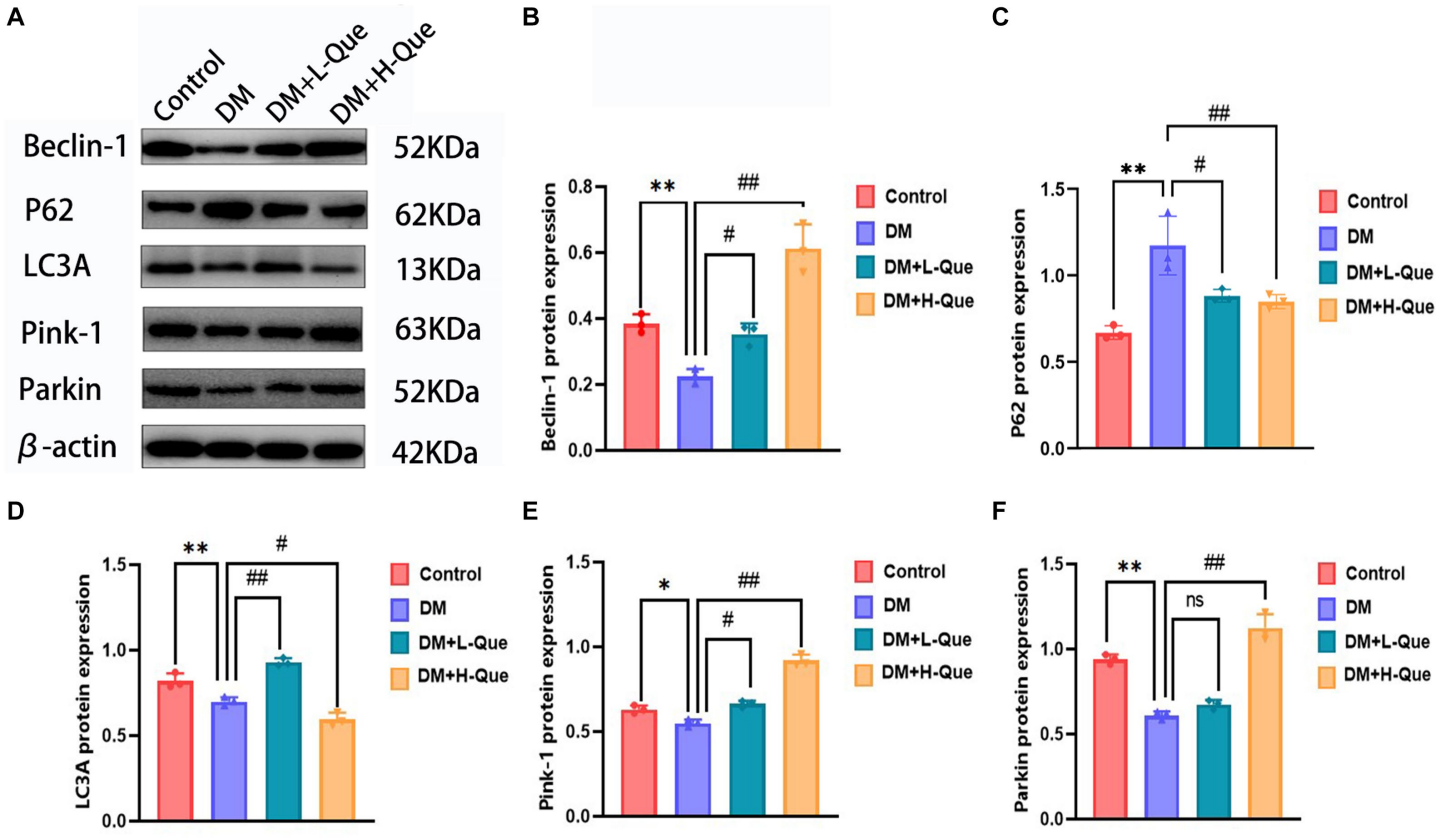
Quercetin promotes autophagy to ameliorate spleen injury in GK rats. **(A)** Western blot gel images of spleen tissues in each group (*n* = 8). **(B–F)** The bar graphs represent the quantification of gel bands (Expression of Beclin1, P62, LC3A, Pink1, and Parkin, mean ± SD). ^**^p < 0.01, comparison with the control group; ^*^p < 0.05, comparison with the control group; ^##^*p* < 0.01, comparison with model group; ^#^*p* < 0.05, comparison with model group, and ns >0.05 shows no difference.

Electrophoretic mobility shift assays revealed that, relative to the control group, GK rats in the model group demonstrated a considerable upregulation of P62 protein expression in their splenic tissues (*p* < 0.01), alongside a significant decrease in the expression levels of Pink-1, LC3A, Beclin-1, and Parkin (*p* < 0.01). Conversely, GK rats in the experimental group receiving a low dosage of quercetin (DM + L-Que) showed a significant reduction in P62 expression (*p* < 0.05), with corresponding significant increases in the expression of Beclin-1, LC3A, Pink-1, and Parkin (*p* < 0.05). Furthermore, GK rats treated with a high dosage of quercetin (DM + H-Que) in comparison to the model group displayed a notable reduction in P62 expression (*p* < 0.01) and significant elevations in the expression of Beclin-1, LC3A, Pink-1, and Parkin (*p* < 0.01). These findings underscore quercetin’s role in safeguarding the spleens of diabetic GK rats through autophagy enhancement, highlighting the dose-dependent effects of quercetin on autophagy (Figure 5).

#### 3.7.3 Analysis of PI3K/Akt/mTOR pathway proteins in the hepatic tissues of GK rats: investigating PI3K, P-PI3K, Akt, P-AKT, mTOR, and P-mTOR expressions

**Figure 6:**
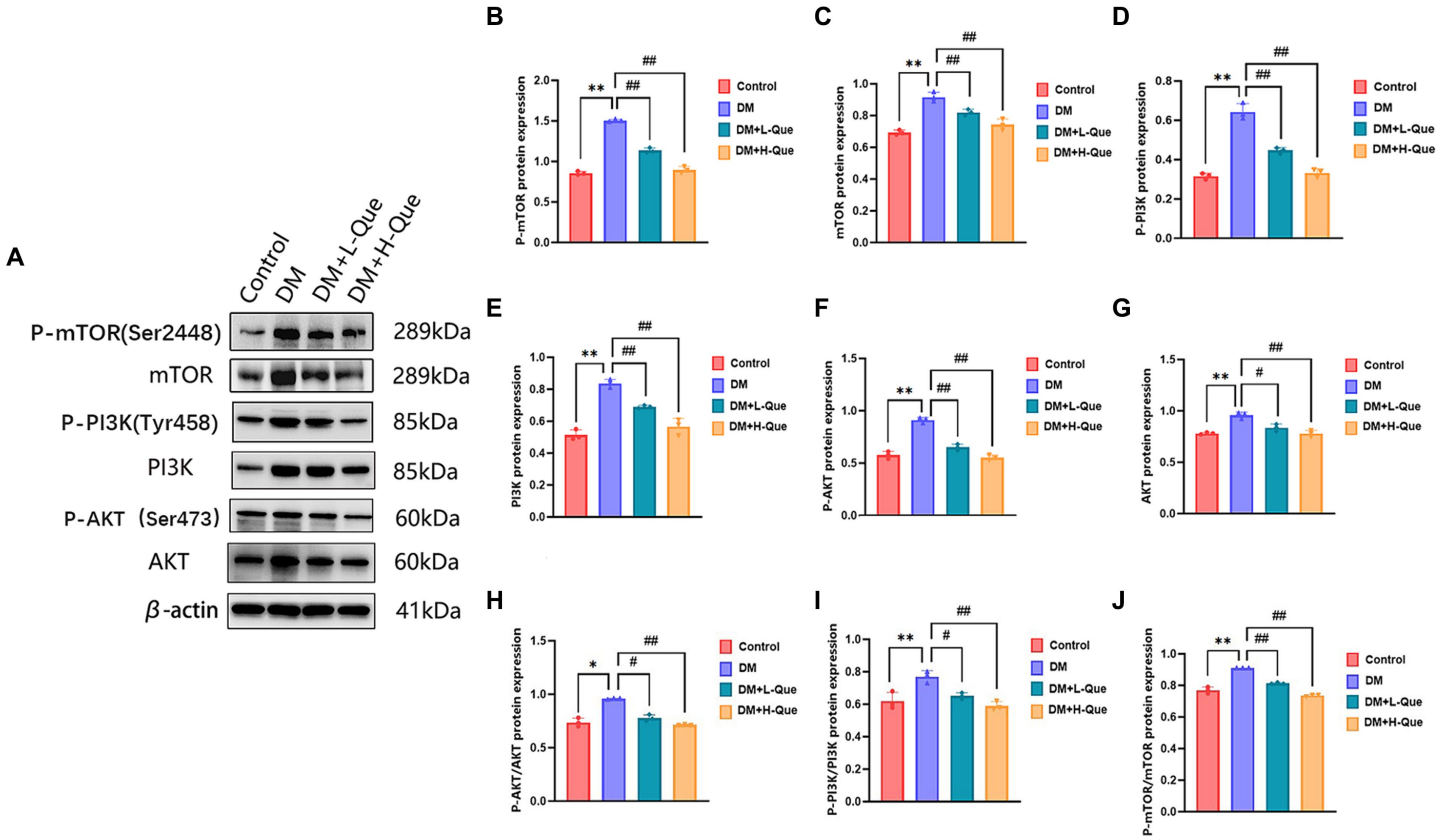
Quercetin regulates the PI3K/AkT/mTOR pathway and ameliorates liver injury in GK rats. **(A)** Western blot gel images of liver tissue in each group (*n* = 8). **(B–J)** The bar graphs represent the quantification of gel bands (Expression of AKT, P-AKT, PI3K, P-PI3K, mTOR, and P-mTOR, mean ± SD). ^**^p < 0.01, comparison with the control group; ^*^p < 0.05, comparison with the control group; ^##^*p* < 0.01, comparison with model group; ^#^*p* < 0.05, comparison with model group.

Western blot analyses demonstrated that, relative to the normal group, the protein expression levels of PI3K, P-PI3K (Tyr458), Akt, P-AKT (Ser473), mTOR, and P-mTOR (Ser2448) in the spleen tissues of model group GK rats were significantly elevated (*p* < 0.01), with the phospho-to-total protein ratios of P-PI3K/PI3K, P-AKT/Akt, and P-mTOR/mTOR experiencing an increase. When compared with the model group, GK rats in the experimental group receiving a low dosage of quercetin (DM + L-Que) showed a substantial decrease in these protein expression levels (*p* < 0.05), along with decreased phospho-to-total protein ratios. Similarly, GK rats administered a high dosage of quercetin (DM + H-Que) exhibited a remarkable reduction in these protein expression index (*p* < 0.01) and in the phospho-to-total protein ratios, illustrating that quercetin exerts a protective effect on the liver of diabetic GK rats by adjusting the PI3K/Akt/mTOR pathway, with the impact of quercetin on autophagy being dosage-dependent (Figure 6).

#### 3.7.4 Differential expression of PI3K/Akt/mTOR pathway proteins in spleen tissues of GK rats among various groups

**Figure 7:**
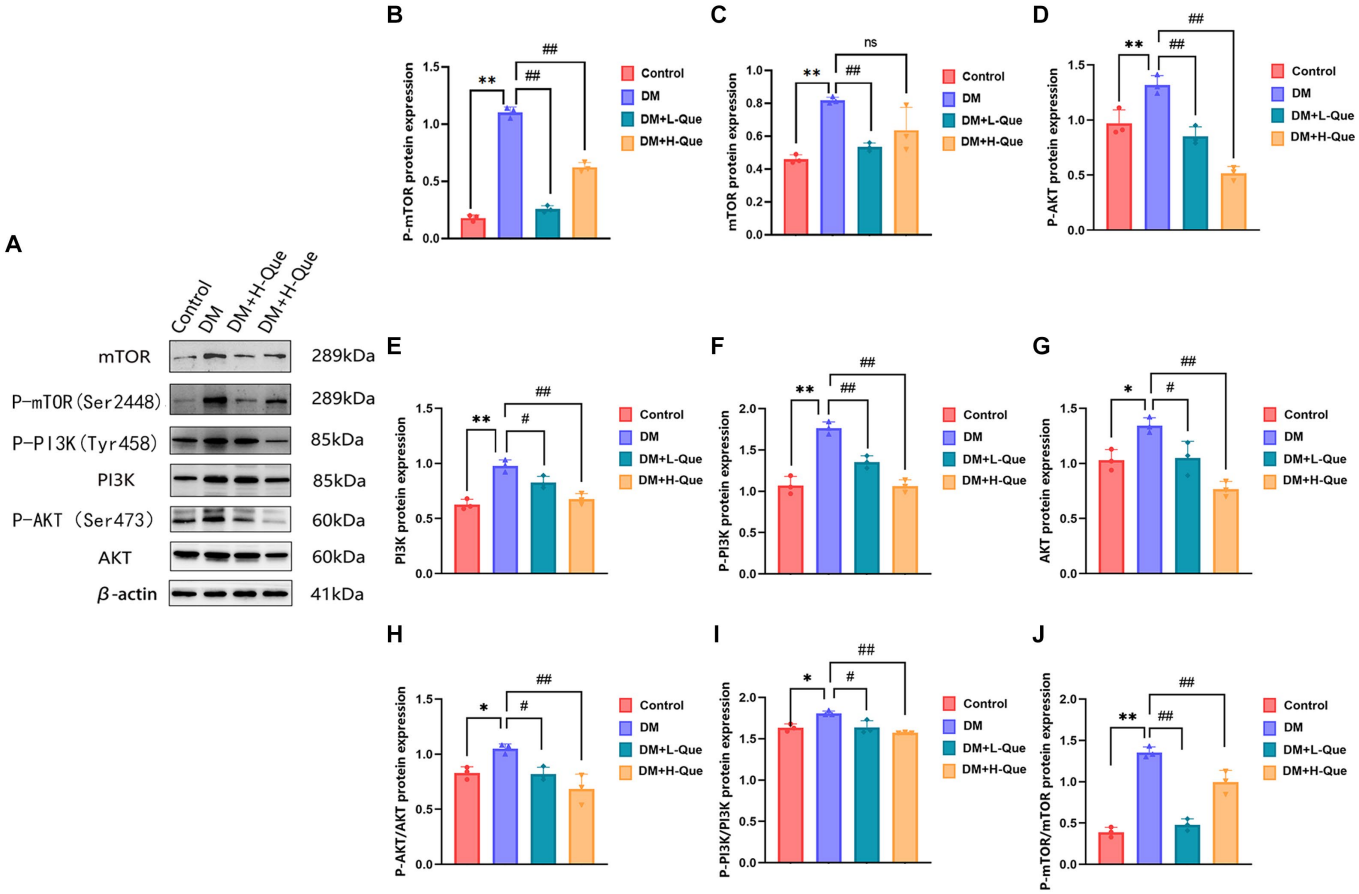
Quercetin can modulate the PI3K/AkT/mTOR pathway and improve spleen injury in GK rats. **(A)** Western blot gel images of spleen tissues in each group (*n* = 8). **(B–J)** The bar graphs represent the quantification of gel bands (Expression of PI3K, P-PI3K, Akt, P-AKT, mTOR, and P-mTOR, mean ± SD). ^**^p < 0.01, comparison with the control group; ^*^p < 0.05, comparison with the control group; ^##^*p* < 0.01, comparison with model group; ^#^*p* < 0.05, comparison with model group, and ns >0.05 shows no difference.

Western blot analysis revealed that relative to the model control group, protein expression index of PI3K, P-PI3K (Tyr458), Akt, P-AKT (Ser473), mTOR, and P-mTOR (Ser2448) in the spleen tissues of GK rats from the model control group experienced a prominent elevation (*p* < 0.01). This was accompanied by increased phospho-to-total protein ratios for P-PI3K/PI3K, P-AKT/Akt, and P-mTOR/mTOR. Conversely, GK rats in the experimental groups receiving low and high doses of quercetin (DM + L-Que and DM + H-Que, respectively) showed a marked decrease in these protein expression levels (*p* < 0.05 for DM + L-Que; *p* < 0.01 for DM + H-Que), with corresponding reductions in the phospho-to-total protein ratios. These findings illustrate quercetin’s role in safeguarding the spleen of diabetic GK rats through PI3K/Akt/mTOR pathway inhibition, highlighting the dosage-dependent nature of quercetin’s effect on autophagy (Figure 7).

## 4 Discussion

Diabetes, a prevalent non-communicable disease, poses a significant threat to global health (25, 26), primarily manifesting through elevated blood glucose levels alongside symptoms including increased hunger, thirst, urination, and unintentional weight loss. It compromises immune function, precipitating adverse effects on multiple organs (27, 28). This condition underscores the interconnectivity and clinical significance of the liver, spleen, and kidneys (29), which play a pivotal role in managing diseases characterized by progressive deterioration and a multitude of complications (30). The complexity of such diseases, often marked by a stealthy onset, relentless progression, extensive organ damage, diverse symptoms, or recurring episodes, presents substantial challenges in prevention and management, drastically diminishing life quality and potentially resulting in disability or fatality. Traditional Chinese Medicine (TCM) (12), anchored in the holistic Zang-Xiang theory, emphasizes the regulation of these organs, offering a robust theoretical framework and invaluable empirical insights for addressing these persistent conditions.

AST and ALT serve as crucial markers in clinical liver function assessments (31), diabetes-induced liver damage results in the release of ALT and AST from hepatic cells into the bloodstream, thereby elevating serum AST and ALT levels. Similarly, TP, CHOL, and UREA are pivotal for evaluating kidney function; their significant elevation indicates kidney impairment due to diabetes, suggesting that diabetes precipitates liver and kidney damage. Quercetin, a widespread natural flavonoid found (32) in various plants such as elderberry, red onion, green pepper, cranberry, lettuce, and spinach, exhibits a broad spectrum of pharmacological benefits, including anti-diabetic (33), antioxidative, anti-fibrotic, anti-inflammatory (34), and antiviral effects. Studies have shown that quercetin significantly mitigates liver inflammation and fibrosis (35), thus ameliorating diabetic nephropathy. Experimental findings reveal that quercetin effectively lowers blood glucose levels, reduces glycated serum proteins, enhances body weight, and diminishes liver and kidney markers in serum, underscoring its beneficial impact on diabetic rats. Consequently, investigating quercetin’s ability to reduce blood glucose and protect against diabetes-induced organ damage holds critical importance for human health maintenance.

Investigation of the protein–protein interaction (PPI) network uncovered that AKT, LC3A, P62, 和 Beclin-1 constitute the four principal targets through which quercetin’s active components exert their therapeutic effects on diabetes. The phosphorylation of AKT (P-AKT) plays a crucial role in the functionality of the AKT signaling pathway (36), influencing critical cellular processes including proliferation, survival, apoptosis, migration, and differentiation.

Autophagy serves as a protective mechanism for cells (37), allowing for the encapsulation and transport of damaged organelles and substances to lysosomes for degradation via autophagic vesicles, thus rejuvenating the cell’s energy resources (37, 38). In the context of liver and spleen damage resulting from diabetes, mitochondrial autophagy levels are found to diminish, whereas an increase in autophagy levels can safeguard against various diseases affecting these organs. LC3, a hallmark protein for autophagy, encompasses LC3A and LC3B as crucial indicators of autophagy levels. Conversely, P62, a specific substrate for autophagy, exhibits an inverse relationship with autophagy levels (39, 40). Beclin-1 plays a pivotal role in initiating autophagy (41) and regulating its activity, while Pink-1 and Parkin are key in mediating mitochondrial autophagy (40). The present study documents a decrease in LC3A/LC3B, Pink-1, Beclin-1, and Parkin protein levels in the model group, alongside an upsurge in p62 expression, suggesting autophagy dysfunction in the liver and spleen of GK rats. Post-treatment, administration of both low and high doses of quercetin elevated the expression levels of LC3A/LC3B, Beclin-1, Pink-1, and Parkin, and diminished p62 expression. Furthermore, it was determined that quercetin alleviates cellular autophagy by downregulating proteins associated with the PI3K/Akt/mTOR pathway, thus mitigating the impact of diabetes on the liver, kidney, and spleen of rats. Consequently, quercetin inhibits the expression of proteins linked to autophagy in diabetic rat liver and spleen tissues via the PI3K/Akt/mTOR pathway, curbing autophagy in these organs and suggesting its potential to rectify autophagy dysfunction in GK rat liver and spleen.

## 5 Conclusion

Diabetes induces substantial damage to the liver, spleen, and kidneys in rats. However, quercetin demonstrates a significant protective effect on these organs in GK rats. This protective mechanism operates through the modulation of autophagy in liver and spleen cells by downregulating the PI3K/Akt/mTOR pathway and affecting autophagic proteins such as LC3A/LC3B, Beclin-1, Pink-1, Parkin, and p62. Notably, higher doses of quercetin amplify this protective effect.

## Data Availability

The raw data supporting the conclusions of this article will be made available by the authors, without undue reservation.

## Footnotes

^1^ https://www.genecards.org/

^2^ https://omim.org/

^3^ https://go.drugbank.com/

^4^ http://db.idrblab.net/ttd/

^5^ https://www.pharmgkb.org/
